# Modeling the Impact of HIV-1 Nucleic Acid Testing Among Symptomatic Adult Outpatients in Kenya

**DOI:** 10.1097/QAI.0000000000003013

**Published:** 2022-05-05

**Authors:** Deven T. Hamilton, Clara Agutu, Joseph B. Babigumira, Elise van der Elst, Amin Hassan, Evanson Gichuru, Peter Mugo, Carey Farquhar, Thumbi Ndung'u, Martin Sirengo, Wairimu Chege, Steven M. Goodreau, Adam Elder, Eduard J. Sanders, Susan M. Graham

**Affiliations:** a Center for Studies in Demography and Ecology, University of Washington, Seattle, WA;; b KEMRI—Wellcome Trust Research Programme, Kilifi, Kenya;; Departments of c Global Health and Pharmacy; and; d Medicine, Global Health, and Epidemiology, University of Washington, Seattle, WA;; e Africa Health Research Institute, Durban, South Africa;; f National AIDS and STI Control Programme, Nairobi, Kenya;; g National Institutes of Allergy and Infectious Diseases, National Institutes of Health, Rockville, MD;; Departments of h Anthropology and Epidemiology; and; i Biostatistics, University of Washington, Seattle, WA; and; j University of Oxford, Headington, United Kingdom.

**Keywords:** HIV prevention, HIV-1 nucleic acid testing, symptomatic testing, PITC, epidemic modeling, Kenya

## Abstract

**Background:**

Up to 69% of adults who acquire HIV in Kenya seek care before seroconversion, providing an important opportunity for early diagnosis and treatment. The Tambua Mapema Plus (TMP) trial tested a combined HIV-1 nucleic acid testing, linkage, treatment, and partner notification intervention for adults aged 18–39 years with symptoms of acute HIV infection presenting to health facilities in coastal Kenya. We estimated the potential impact of TMP on the Kenyan HIV epidemic.

**Methods:**

We developed an agent-based network model of HIV-1 transmission using TMP data and Kenyan statistics to estimate potential population-level impact of targeted facility-based testing over 10 years. Three scenarios were modeled: standard care [current use of provider-initiated testing and counseling (PITC)], standard HIV rapid testing scaled to higher coverage obtained in TMP (scaled-up PITC), and the TMP intervention.

**Results:**

Standard care resulted in 90.7% of persons living with HIV (PLWH) knowing their status, with 67.5% of those diagnosed on treatment. Scaled-up PITC resulted in 94.4% of PLWH knowing their status and 70.4% of those diagnosed on treatment. The TMP intervention achieved 97.5% of PLWH knowing their status and 80.6% of those diagnosed on treatment. The percentage of infections averted was 1.0% (95% simulation intervals: −19.2% to 19.9%) for scaled-up PITC and 9.4% (95% simulation intervals: −8.1% to 24.5%) for TMP.

**Conclusion:**

Our study suggests that leveraging new technologies to identify acute HIV infection among symptomatic outpatients is superior to scaled-up PITC in this population, resulting in >95% knowledge of HIV status, and would reduce new HIV infections in Kenya.

## Abstract 2

Supplemental Digital Content is Available in the Text.

## INTRODUCTION

Data on progress toward the UNAIDS 90–90–90 targets show that in 2019, approximately 81% of persons living with HIV infection (PLWH) globally were aware of their status, 67% were on antiretroviral treatment (ART), and 59% had achieved viral suppression.^1^ Although HIV incidence has declined by 38% in Eastern and Southern Africa since 2010, HIV test coverage is estimated at 87%, still below the 90% target.^1^ In response to persistent disease burden, UNAIDS set new targets to “end the HIV epidemic” in 2014,^2^ stipulating that 95% of all PLWH will be diagnosed, 95% of those diagnosed will be on ART, and 95% of those taking ART will have achieved virologic suppression by 2030.^2^ Because HIV testing is the gateway to both the HIV care and prevention cascades, it is critical that a variety of differentiated testing strategies be deployed to meet these ambitious targets.

HIV testing strategies encompass diverse activities at numerous sites, with health facilities being critical sites for HIV diagnosis among patients seeking care. In a systematic review published in 2015, Sharma et al^3^ found that while community HIV testing and counseling had higher coverage, provider-initiated testing and counseling (PITC) had higher uptake and resulted in the highest proportion of new diagnoses among individuals with CD4 counts ≤350.^3^ They concluded that improving PITC coverage could be an important adjunct to community-based testing strategies in African settings. Less is known about the diagnosis of acute HIV infection (AHI) in health care settings, although algorithms targeting patients more likely to have AHI have been published and undergone limited validation.^4–6^ Because adults with AHI often seek care for symptoms of infectious illness,^7,8^ an approach targeting patients with such symptoms with a testing strategy capable of detecting both acute and chronic HIV infection could increase the yield of facility-based testing and contribute importantly to interrupting HIV transmission.

The Tambua Mapema (“Discover Early”) Plus study (NCT03508908), conducted in Kenya (2017–2020), evaluated the yield of an HIV-1 nucleic acid testing intervention to diagnose acute and chronic HIV infections.^9^ The trial enrolled adult patients aged 18–39 years who had no prior HIV diagnosis and scored ≥2 on a risk score with the following scoring: age 18–29 years, fever, fatigue, body pains, diarrhea, sore throat (each 1 point), or genital ulcer disease (3 points). Participants were first enrolled during an observation period in which provider testing behavior and new HIV diagnoses were documented. This period was followed by an intervention period during which participants were offered HIV-1 nucleic acid testing using point-of-care Xpert HIV-1 Qual (Cepheid, Sunnyvale, CA) testing, followed by standard HIV rapid tests to distinguish acute from chronic infections. Individuals newly diagnosed in the intervention period were immediately linked to ART initiation and offered partner notification services (PNS); their partners were offered ART or pre-exposure prophylaxis (PrEP) depending on HIV status.^10^

Mathematical modeling has been used to evaluate the potential impact of diverse HIV interventions on HIV transmission, morbidity, mortality, and other outcomes^11–15^ and to prioritize resource allocation.^16^ The objective of this study was to model the potential impact of the HIV-1 nucleic acid testing, linkage, treatment, and PNS interventions, as delivered in the Tambua Mapema Plus (TMP) trial, on the Kenyan HIV epidemic. Specifically, our aim was to evaluate the TMP intervention's impact on the first 2 UNAIDS 95-95-95 targets by modeling the proportion of PLWH who would know their status and the proportion of those diagnosed who would be on treatment. We also estimated the number of infections averted and reductions in HIV prevalence if the intervention was the standard of care in Kenyan health facilities. Given prior calls in the literature to increase PITC, we also modeled the potential impact of an alternative strategy in which PITC using standard rapid antibody tests was scaled up to match the level of testing in the TMP intervention, without the additional components (ie, nucleic acid tests, expedited linkage, or enhanced PNS) of the TMP intervention, thereby isolating just the impact of improved testing for chronic infections.

## METHODS

We developed a stochastic, agent-based network model to simulate the HIV epidemic in Kenya. The model was built on the EpiModel (http://epimodel.org/) and *statnet* (http://statnet.org/) software platforms.^17,18^ The EpiModel HIV R package was used to incorporate the relevant HIV-specific epidemiology for this study (https://github.com/statnet/EpiModelHIV/tree/Kenya-TM). Similar models have been used previously to model HIV and STI interventions in other contexts.^13,14,19^ Full details of the model and parameters are presented in the technical appendix (see Appendix, Supplemental Digital Content 1, http://links.lww.com/QAI/B900), but we provide a general overview here.

The simulation included 10,000 heterosexual adults aged 18–39 years, with the same sex and age composition as reported in *Kenya Facts and Figures 2015*.^20^ Simulations were run more than 10 years in 1-week discrete time intervals. Individuals could engage in relationships of 3 types (main, casual, and one-time). Formations of new relationships were conditional on current relationships, and each relationship had type-specific within-partnership behaviors (eg, HIV status disclosure, condom use, and coital frequency) and duration. Demographic changes in the population included new individuals entering the simulation at age 18 years, aging, mortality, and exit because of mortality or aging out of the population. Intrahost epidemiology included the natural progression of disease within PLWH in the absence of clinical intervention. The main component of disease progression explicitly modeled for this study was HIV viral load, which controlled interhost epidemiology (HIV transmission rates) and disease progression. Changes in viral load were a function of time since infection and ART status. The clinical epidemiological processes in the model after infection included diagnosis, linkage to care, treatment initiation/reinitiation, adherence, and HIV viral suppression. Transitions along the continuum of care occurred stochastically. The model was parameterized using data from the TMP study and Kenya-specific parameters drawn from the literature.

Three main scenarios were modeled: standard care (current use of PITC), scaled HIV rapid testing (scaled-up PITC), and the TMP intervention.^9^ The parameters that define the differences in the simulation under each of the 3 main scenarios are summarized in Table 1. The simulation included multiple routes to HIV testing to capture different ways people come to test and the different components of the TMP intervention. These routes to testing were divided into 2 categories, which we will refer to as clinical and background testing.

**TABLE 1.** Testing, Treatment, and Prevention Parameters Under Standard-of-Care, Scaled-Up PITC and the TMP Intervention, With Differences Between Modeled Scenarios in Bold Italics Within Cells

Parameter	Standard care: PITC at Current Rates	Scaled-Up PITC	TMP Intervention
Probability of experiencing AHI-like symptoms and presenting to a health care facility regardless of HIV status*	0.0006/wk	0.0006/wk	0.0006/wk
Probability of experiencing AHI symptoms and presenting to a health care facility given recent HIV infection†	0.325/wk for 3 weeks after infection	0.325/wk for 3 weeks after infection	0.325/wk for 3 weeks after infection
Probability of being tested by a clinician after presenting to a health care facility with AHI-like symptoms‡	25.6%	***94.9%***	***94.9%***
HIV test type	Two rapid antibody tests	Two rapid antibody tests	***Nuclear acid test (GeneXpert®*** ***HIV-1 Qual) followed by 2 rapid antibody tests if positive***
Test window period	3 wk	3 wk	***1 week***
Partner services eligibility§	Partners from the past 12 months	Partners from the past 12 months	***Partners from the past 12 weeks if index case acute infection; partners from the past 12 months if index case chronic infection***
Probability a partner was tested§	0.023/week for 6 weeks of follow-up	0.023/week for 6 weeks of follow-up	***0.169/week for 6 weeks of follow-up***
Tests used for partner services§	Two rapid antibody tests	Two rapid antibody tests	***Nuclear acid test (GeneXpert®*** ***HIV-1 Qual) followed by 2 rapid antibody tests if positive***
Probability of initiating ART after diagnosis	0.159/week for 8 wk	0.159/week for 8 wk	***0.31/week for 6 weeks if diagnosed in a clinic diagnosis based on AHI symptoms;*** ***0.159/week for 8 weeks if diagnosed by background testing***
Probability of discontinuing ART	0.002/week	0.002/week	0.002/week
Probability of reinitiating ART after discontinuation or initiating ART if ART was not initiated after within 8 weeks of diagnosis	0.01/week	0.01/week	0.01/week

* Sensitivity analyses evaluated the impact of a lower probability of experiencing AHI-like symptoms and presenting to a health care facility regardless of HIV status; values (0.002, 0.009).

† Sensitivity analyses evaluated the impact of a lower proportion (57.5%) of individuals newly infected with HIV experiencing AHI-like symptoms and seeking treatment at a health care facility which changed the rate to 0.248/week.

‡ Sensitivity analyses evaluated the impact of alternative probabilities of being tested by a clinician after presenting to a health care facility with AHI-like symptoms including a more modest improvement to 45% and a maximum possible 100%.

§ Sensitivity analyses evaluated the impact of TMP in the absence of changes to partner services from the current standard of care.

### Standard Care

Clinical testing included PITC of anyone who sought care at a health facility in response to symptoms that met the case definition for testing in the TMP intervention. The probability an individual experienced AHI symptoms (regardless of HIV status) and presented at a health facility was 0.0006/week based on health histories reported in the TMP study. Individuals actually experiencing AHI also sought care in response to their symptoms at a constant rate (0.325/week) that reproduced the reported 69% seeking care before seroconversion^7^ which occurred 3 weeks after infection in the simulation.

The probability of being tested when seeking care was 25.6% in the PITC scenario, the rate observed during the observation period of the TMP study. Under standard care testing used 2 rapid antibody tests, Determine from Abbott Laboratories and First Response from Premier Medical Corporation. Sensitivity and specificity were based on the published values. In the simulation, these tests could detect antibodies 3 weeks after HIV acquisition. Partners from the prior year were eligible for PNS and had a constant 0.023 hazard of being tested over the subsequent 6 weeks based on a recent study of PNS in Kenya.^21^ Partners who tested in the PITC and scaled-up PITC scenarios were tested with rapid antibody tests.

### Scaled-up PITC

In the scaled-up PITC scenarios, the probability that individuals received a test was increased to 94.9% to match the level of testing achieved in the intervention arm of the TMP study. All other parameters of this model were the same as standard care.

### TMP Intervention

In the TMP scenario, symptomatic individuals presenting at a health facility had a 94.9% probability of being tested, based on the proportion of participants tested in the intervention arm.^10^ Testing used the GeneXpert HIV-1 Qual test (sensitivity = 0.989, specificity = 1.0). In the simulations, the HIV-1 Qual test could diagnose infection after 1 week. Partners from the prior year or prior 12 weeks were eligible for PNS if the index case was a chronic or acute infection, respectively. Partners had a weekly hazard of 0.169 of testing as part of PNS, based on the 67% of partners tested in the PNS arm of the study by Cherutich et al.^21^ Partners were tested with the HIV-1 Qual test which we term “enhanced PNS.” Given the lack of data and overall low level of PrEP use in Kenya, PrEP was not included in the simulation.

### Background Testing

Community-based testing and PITC in the absence of AHI symptoms are common in Kenya.^3,22^ We therefore included in all 3 modeled scenarios testing for reasons other than symptomatic illness, which was not altered by either scaled-up PITC or the TMP intervention. We back-calculated age-specific and sex-specific rates of testing to match estimates from the 2014 Kenya Demographic and Health Survey.^23^ Background testing used rapid antibody tests, consistent with the standard of care in Kenya, and was not linked to PNS, consistent with the currently low uptake of this intervention in the study area.

### HIV Treatment

After diagnosis, individuals had a weekly hazard of 0.159 of starting ART for 8 weeks, such that 75% of those diagnosed were on treatment within 8 weeks, consistent with the rate of ART uptake in Kenya reported by Odeny et al.^24^ This uptake hazard applied to background testing and clinical testing under standard and scaled-up PITC. In the TMP study, 89.2% of individuals who tested positive in the intervention period were on ART by 6 weeks^10^; thus, those diagnosed in health facilities in the TMP scenario had a higher weekly hazard (0.31) of starting ART for 6 weeks after diagnosis. After ART initiation, individuals had a constant 0.002 hazard of discontinuation, such that 90% of those who initiated remained on ART at 12 months, consistent with reported retention rates in Kenya.^25^ After ART discontinuation, individuals had a weekly hazard of 0.01 of reinitiating ART; this same hazard was also used to determine ART initiation for those who did not initiate ART in 8 or 6 weeks after diagnosis in the PITC and TMP interventions, respectively. This hazard of reinitiation was calculated as a tuning parameter using simulation, to match the 75% overall ART coverage among adults and young people reported in Kenya,^25^ given the initiation and discontinuation hazards for ART under standard care.

Once on ART, 23% of cases achieved partial viral suppression (Vl: 3.5 log_10_) while 77% of cases achieved full viral suppression (Vl: 2.75 log_10_). The proportions achieving partial and full suppression were set to match the reported 77% of PLWH taking ART in Kenya achieving viral suppression.^25^

### Sensitivity Analyses

In addition to the 3 primary scenarios outlined above, we conducted several sensitivity analyses. We simulated 45% and 100% uptake of the TMP intervention to approximate a more conservative real-world uptake in the absence of a clinical trial and the maximum possible impact, respectively. We also simulated the impact of the TMP intervention without enhanced PNS, given the challenges of contact tracing in resource limited settings. We repeated the primary analyses using a lower (more conservative) probability (57.5%) of care seeking because of AHI symptoms based on the lower bound of the 95% confidence interval of our estimate from the study by Sanders et al.^7^ The lower 57.5% estimate is also a midway between the estimate by Sanders et al which was from a study conducted in Kenya and a lower 53% estimate from a study in the United Kingdom^8^ where symptoms are likely to be less severe because of the predominant HIV subtypes. Finally, we repeated the 3 main analyses with higher (0.009) and lower (0.002) probabilities of presenting at a health facility with AHI symptoms because of uncertainty in our estimate of the frequency with which individuals experience these symptoms and seek care as a result.

## RESULTS

### Testing Outcomes

**FIGURE 1.**
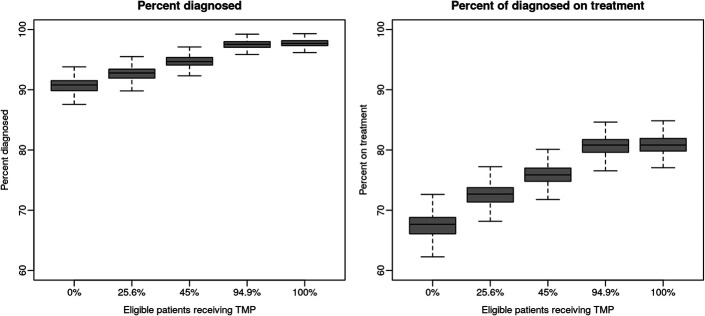
Percentage of HIV-1–infected individuals aware of their status and the percent aware of their status on treatment in Kenya over 10 years with current rates of provider-initiated treatment and counseling compared with 4 levels of Tambua Mapema Plus intervention uptake.

In the standard PITC scenario, 88,387 tests were administered on average over 10 years, of which 7963 (9.0%) were clinical tests. In this scenario, 605 individuals [95% simulation interval (SI): 694–529] were newly infected with HIV, of whom 69% (417) presented at a clinic with AHI symptoms and 25.9% (108) were referred for testing. Of all PLWH, 90.7% (95% SI: 88.3%–92.8%; Table 2) were aware of their infection status, consistent with current estimates from Kenya.^1^ In the scaled-up PITC scenario, improving the rate of testing to 94.9% increased the number of clinical tests from 7963 to 28,724, which accounted for 26% of all testing. The rate of care seeking among those newly infected remained consistent at 69% (411), but 94.9% (390) of AHI cases presenting at clinics were referred for testing. Scaling-up PITC increased the percent of PLWH who knew their status to 94.4% (95% SI: 92.7%–96.1%), just shy of the UNAIDS 95-95-95 goals. Finally, in the TMP intervention scenario, increased testing rates coupled with HIV-1 Qual testing, expedited linkage, and enhanced PNS resulted in a similar number of clinical tests administered (28,646) but increased the proportion of PLWH who were aware of their status to 97.5% (95% SI: 96.3%–98.6%), reaching the new UNAIDS targets for the first step in the HIV care continuum (Fig. 1). Although in the standard care PITC scenario, only 67.5% (95% SI: 64.1%–70.6%) of diagnosed cases were on ART, the TMP intervention, which included expedited linkage to care, improved ART coverage by 19.4%, to 80.6% (95% SI: 77.8%–83.2%).

**TABLE 2.** Simulated Impact of the Tambua Mapema Plus Intervention on the HIV Care Continuum

Intervention	Percent of Symptomatic Individuals tested (%)	Mean Percent Diagnosed and 95% SIs	Mean Percent of Diagnosed on Treatment and 95% SIs
PITC at current rates	25.6	90.7, (88.3–92.8)	67.5, (64.1–70.6)
TMP*	25.6	92.7, (90.7–94.5)	72.5, (69.3–75.2)
	45.0	94.7, (93.1–96.3)	75.9, (73–78.5)
	94.9	97.5, (96.3–98.6)	80.6, (77.8–83.2)
	100	97.7, (96.7–98.8)	80.9, (78–83.8)
Increased PITC only†	45.0	91.8, (90–93.6)	68.2, (65.4–71.5)
	94.9	94.4, (92.7–96.1)	70.4, (66.9–73.5)
	100	94.5, (92.9–96.1)	70.7, (67.5–74.3)
TMP w/o PNS‡	94.9	96.8, (95.4–97.8)	79.8 (77.2–82.4)

* The TMP intervention consisted of HIV-1 RNA testing followed by standard rapid HIV tests to distinguish acute from prevalent infection. Newly diagnosed participants were immediately linked to care and offered PNS by a dedicated intervention team.

† Increased PITC using standard rapid HIV tests will not diagnose acute infection. Newly diagnosed participants were linked to care at the same facility and not consistently offered PNS.

‡ The TMP w/o PNS intervention consisted of HIV-1 RNA testing followed by standard rapid HIV tests to distinguish acute from prevalent infection. Newly diagnosed participants were immediately linked to care but were not consistently offered PNS.

### Infections Averted

**FIGURE 2.**
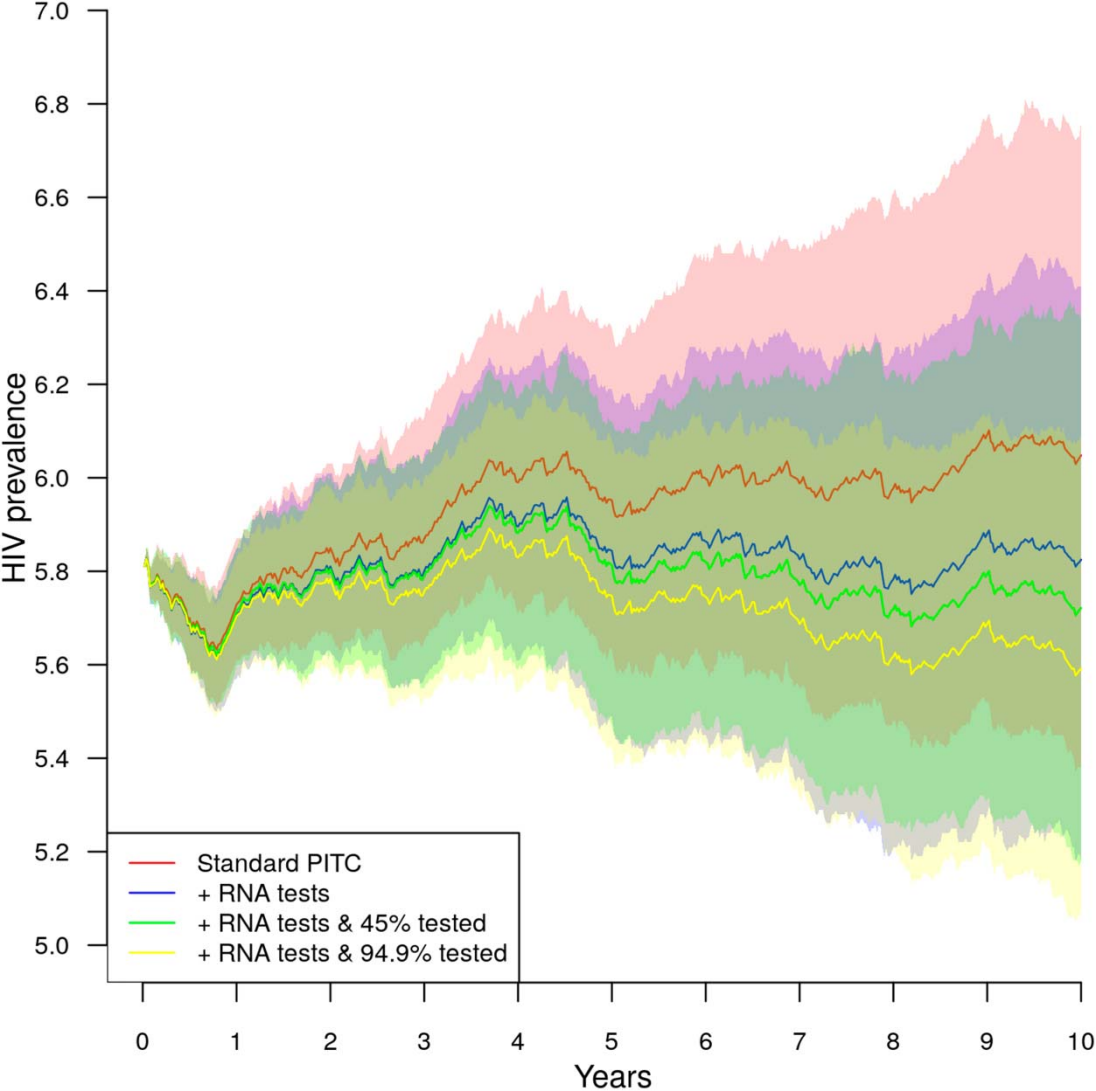
HIV-1 prevalence in Kenya over 10 years with current rates of provider-initiated treatment and counseling compared with 3 levels of Tambua Mapema Plus intervention uptake.

Scaling-up PITC alone averted just 1% (95% SI: −19.2% to 19.9%) of infections over 10 years relative to the current level of PITC and had almost no impact on HIV prevalence (Table 2). Increasing testing among individuals experiencing AHI symptoms did reduced the time from infection to diagnosis substantially. The average duration from infection to diagnosis with scaled-up PITC was 31.5 weeks (95% SI: 28.7–34.2) compared with 48 weeks (95% SI: 44.7–51.8) in the PITC scenario. The TMP intervention averted 9.4% (95% SI: −8.1% to 24.5%) of infections and reduced the prevalence of HIV in the population from 6.1% (95% SI: 5.4%–6.8%) to 5.6% (95% SI: 5.1%–6.1%) (Fig. 2). The TMP intervention also reduced HIV incidence by 20%, from 0.67 per 100 person-years at risk under standard PITC to 0.53 per 100 person-years at risk. The average duration from infection to diagnosis was just 16.6 weeks (95% SI: 14.4–18.7) compared with 48 weeks (95% SI: 44.7–51.8) in the PITC scenario, a reduction of 65%.

### Sensitivity Analyses

In our first sensitivity analysis, increasing uptake from 25.6% to 45% and to a maximum 100% produced improvement in both the fractions of PLWH who were aware of their HIV status and the fraction of those diagnosed who were on ART roughly linearly with testing coverage in both the scaled-up PITC and TMP scenarios, but the TMP intervention had a larger impact at each level of testing (Tables 2 and 3). Implementing the TMP intervention without the improvement in PNS participation and HIV-1 Qual testing for recent partners of acute patients achieved improvement in diagnosis and linkage to care that were similar to the full TMP intervention; 96.8% compared with 97.5% diagnosed and 79.8% on treatment compared with 80.6%. The exclusion of enhanced PNS also did not significantly change the percent of infections averted. However in the absence of enhanced PNS, the number of weeks between infection and diagnosis increased by 3 months on average.

**TABLE 3.** Simulated Impact of the Tambua Mapema Plus Intervention on HIV Outcomes

Intervention	Percent of Symptomatic Individuals Tested (%)	Mean Percent of Infections Averted and 95% SI	Mean Number of Infections Averted per 100K Person yr at Risk and 95% SI	Mean HIV Prevalence and 95% SI	Mean Number of wk from Infection to Diagnosis and 95% SI	Mean HIV Incidence per 100 Person yr at Risk and 95% SI
PITC at current rates	25.6	#N/A	#N/A	6.1, (5.4–6.8)	48, (44.7–51.8)	0.67, (0–1.68)
TMP*	25.6	4, (−14.5 to 21.2)	29.6, (−81.7 to 150.5)	5.8, (5.2–6.4)	37.7, (34.2–41.4)	0.60, (0–1.66)
	45.0	6.3, (−12.3 to 22.4)	44.4, (−69.8 to 160.8)	5.7, (5.2–6.3)	29.5, (26.5–32.9)	0.56, (0–1.66)
	94.9	9.4, (−8.1 to 24.5)	64.1, (−45.5 to 179.8)	5.6, (5.1–6.1)	16.6, (14.4–18.7)	0.53, (0–1.65)
	100	9.9, (−7.2 to 25.8)	67.3, (−46.8 to 180.7)	5.6, (5.1–6.1)	15.8, (13.9–18)	0.47, (0–1.65)
Increased PITC only†	45.0	0.2, (−20.1 to 18.1)	5.9, (−119.1 to 122.9)	6, (5.4–6.7)	42.4, (38.7–45.7)	0.57, (0–1.66)
	94.9	1, (−19.2 to 19.9)	10.7, (−117 to 138.7)	6, (5.3–6.6)	31.5, (28.7–34.2)	0.59, (0–1.66)
	100	2.3, (−16.8 to 21.5)	18.7, (−96.8 to 159.4)	5.9, (5.3–6.6)	30.5, (27.7–33.6)	0.60, (0–1.66)
TMP w/o PNS‡	94.9	9.5, (−1.3 to 20.3)	60.7, (−8.6 to 129.5)	5.6, (5.1–6.1)	19.7, (17.5–22.9)	0.54, (0–165)

* The TMP intervention consisted of HIV-1 RNA testing followed by standard rapid HIV tests to distinguish acute from prevalent infection. Newly diagnosed participants were immediately linked to care and offered PNS by a dedicated intervention team.

† Increased PITC using standard rapid HIV tests will not diagnose acute infection. Newly diagnosed participants were linked to care at the same facility and not consistently offered PNS.

‡ The TMP w/o PNS intervention consisted of HIV-1 RNA testing followed by standard rapid HIV tests to distinguish acute from prevalent infection. Newly diagnosed participants were immediately linked to care but were not consistently offered PNS.

Sensitivity analyses (see Tables S1 and S2, Supplemental Digital Content 1, http://links.lww.com/QAI/B901) showed that our findings were robust to error in our estimate of care-seeking after HIV infection. In the standard care PITC scenario, using a lower probability (57.5%) of seeking care compared with the base case (69%) only reduced the percent diagnosed from 90.7% (95% SI: 88.3%–92.8%) to 90.4% (95% SI: 88.3%–92.5%). The impact of this change was similarly small in the TMP intervention scenario, reducing the percent diagnosed from 97.5% (95% SI: 96.3%–98.6%) to 96.7% (95% SI: 95.4%–97.9%). The small change in diagnosis using these different estimates for the probability of care seeking was largely driven by the overall high rate of testing, which meant that most individuals were eventually tested regardless of how they responded to a particular instance of AHI symptoms. A larger impact was observed on the infections averted, which declined from 9.4% (95% SI: −8.1 to 24.5) to 8.1% (95% SI: −8.9 to 23.2), and the average time from infection to diagnosis, which increased from 16.6 weeks (95% SI: 14.4–18.7) to 20.4 weeks (95% SI: 17.9–23.2) in the TMP intervention condition. Varying the rate of background care seeking from 0.006 to 0.002 or 0.009 similarly had minimal effect on outcomes.

## DISCUSSION

Since the peak of the global HIV epidemic in the 1990s, great strides have been made in diagnosing PLWH, ensuring prompt treatment, and interrupting HIV transmission, but new infections in 2019 (ie, 1.7 million) still exceeded the UNAIDS target of 500,000 by 2020.^1^ Unfortunately, it is becoming ever more difficult to improve outcomes, particularly at the population level. The latest estimates suggest that 87% of the Kenyan population has received at least 1 HIV test,^1^ and younger cohorts are continuing to test at greater frequency than their predecessors, driving testing coverage even higher. With such high levels of testing already in place and prevalence approaching just 5%, increasing the frequency of testing across the entire population will require millions of additional tests, most of which will be negative.

Because most of the Kenyan adults with AHI seek care for symptoms of infectious illness,^7^ there is an opportunity early after HIV-1 acquisition to target testing specifically to those individuals most likely to be infected at a point soon after they acquire infection. In our simulation of standard care PITC at current levels, although 90% of individuals knew their status, it took on average 48 weeks from the time they were infected until they were diagnosed. This suggests that there is significant room for improvement in the speed of diagnosis. By leveraging a symptoms-based algorithm and targeting adults aged 18–39 years (the age cohort with the highest incidence) who presented at a health facility with AHI symptoms, we were able to dramatically reduce the estimated time between infection and diagnosis, which declined by 65%.

Earlier detection reduced the amount of person-time spent undiagnosed, which in turn increased the fraction of PLWH who are diagnosed (97.5% in our simulation) and improved the first step of the HIV continuum of care. Earlier detection also allowed for more rapid linkage to care, which improves patient outcomes and interrupts at least some secondary HIV transmission, thereby improving population-level outcomes (ie, prevalence). One of the core components of the UNAIDS strategy to end the HIV epidemic is treatment as prevention (TASP).^26^ Thus, a key component of the TMP intervention was expedited linkage to care after diagnosis. By reducing the time both from infection to diagnosis and from diagnosis to ART initiation, the TMP intervention increased the proportion of PLWH who were diagnosed and on treatment (the second step of the HIV care continuum) by 28.4%. Because individuals on treatment were less likely to transmit HIV to their sexual partners, this resulted in a decline in the incidence rate of 20%. This decline in incidence is similar to that reported for PopART, a highly intensive community-based universal testing and treatment program,^27^ suggesting that a potentially more efficient clinic-based approach may be able to achieve the same impact. These results also suggest that using the TMP intervention to improve TASP coverage could potentially be sufficient to achieve the UNAIDS goals. Improved PNS as part of the TMP intervention did not significantly change population-level epidemic outcomes, but it did account for a 3-month reduction in the time from infection to diagnosis, 10% of the overall improvement in early detection. PNS may become more important as prevalence declines and it becomes more difficult to efficiently identify PLWH.

The TMP protocol included a PrEP component for negative partners of newly diagnosed cases.^9^ PrEP is another pillar of the UNAIDS effort to end the HIV epidemic.^26^ However, owing to very small numbers in the TMP PrEP cohort and low PrEP uptake in Kenya to date,^25,28^ PrEP was not included in the simulations. Thus, all the improved outcomes shown here reflect exclusively the impact of a more targeted approach to HIV testing and TASP. Given the high cost of PrEP and challenges with PrEP uptake and adherence in many African studies to date,^28–30^ it may be more cost-effective to focus on targeting and efficiency improvements in diagnosis and treatment rather than funding large-scale PrEP programs. However, we are unable to say to what additional extent HIV incidence may have declined with increased PrEP, either within serodiscordant couples or delivered to specific key populations. Future simulation studies may provide greater insight into the additional contributions that scaling-up PrEP could make to reducing HIV transmission in Kenya. Nonetheless, our current analysis demonstrates that significant improvements in HIV outcomes can be made in the absence of PrEP.

Limitations of this study include the exclusion of specific key populations such as adolescents, men who have sex with men, commercial sex workers, and injection drug users, all of whom may be important targets for intervention,^15^ no specific interventions targeting retention in care or ART adherence and no drug resistance. The 19.4% improvement in ART coverage seen in the modeled TMP intervention was exclusively a result of the improved linkage to care reported in the TMP study. This suggests that expedited linkage alone can have a big impact on the epidemic. It is possible that interventions to improve retention in care, such as SMS reminders or differentiated care models,^31,32^ could improve retention in care enough to increase the proportion of those diagnosed who are on treatment in Kenya to the 95% UNAIDS target. In addition, interventions to improve ART adherence or changes to more effective regimens that increase the proportion of treated individuals who are virally suppressed was outside the scope of the TMP intervention and this modeling study. A TASP approach combining the TMP intervention with an effective intervention to promote viral suppression could effectively shut down a substantial proportion of transmission.

Another limitation of this analysis is that it does not include a cost-effectiveness analysis, which will be an important part of evaluating the feasibility of an intervention such as TMP, which replaced standard, low-cost HIV rapid tests with relatively costly nucleic acid tests and therefore certainly increase the cost of testing. We are in the process of completing a cost-effectiveness analysis of the TMP intervention. Finally, validation of agent-based microsimulation models can be difficult to perform in the traditional sense because the events being modeled have not yet happened, and the population represented in the model is hypothetical and stylized to address a specific question. The model used here has strong theoretical^17,33–36^ and empirical foundations^37–47^ that support its use here. Despite these limitations, these types of models are becoming the standard for epidemic modeling because they are able to capture far more complexity and heterogeneity than other modeling methods.

In conclusion, our study suggests that leveraging new technologies such as point-of-care nucleic acid testing to diagnose AHI among symptomatic adult outpatients, combined with programmatic improvements such as expedited linkage to care and enhanced PNS aimed at reducing HIV transmission, are superior to scaled-up PITC in this population, resulting in >95% knowledge of HIV status, and would reduce new HIV infections in Kenya. In addition, a 2021 study conducted in Eswatini evaluating the use of a predictors risk score and viral load testing to identify acute and early HIV concluded that such an approach to diagnosis and care seems possible in resource-limited settings.^48^ Their findings suggest that the TMP intervention is potentially feasible and impactful. Missing HIV diagnoses when patients are most infectious is no longer acceptable if we truly wish to end the HIV epidemic.

## Supplementary Material

SUPPLEMENTARY MATERIAL
