# Supplementary material for: Modeling the Impact of HIV-1 Nucleic Acid Testing Among Symptomatic Adult Outpatients in Kenya
Source: J Acquir Immune Defic Syndr. 2022 May 5;90(5):553–61. doi: 10.1097/QAI.0000000000003013 (PMC9259037; doi:10.1097/QAI.0000000000003013)
Supplement: SUPPLEMENTARY MATERIAL [file qai-90-553-s001.docx]

**Table S1. Sensitivity of model predictions to assumptions about the weekly probability of experiencing symptoms compatible with acute HIV and seeking treatment**

| Intervention | % of eligible patients tested | Probability of symptoms and seeking treatment | Percent diagnosed | Percent on Treatment | Percent of infections averted | HIV prevalence |
| --- | --- | --- | --- | --- | --- | --- |
| PITC at current rates | **25.6%** | **0.002** | 90.6, 95% SI: (88.4, 92.7) | 67.4, 95% SI: (63.7, 71) | #N/A | 6, 95% SI: (5.4, 6.5) |
|  |  | **0.006** | 90.7, 95% SI: (88.3, 92.8) | 67.5, 95% SI: (64.1, 70.6) | #N/A | 6.1, 95% SI: (5.4, 6.8) |
|  |  | **0.009** | 90.6, 95% SI: (88.5, 92.7) | 67.5, 95% SI: (64, 70.8) | #N/A | 6, 95% SI: (5.4, 6.7) |
| TMP^a^ | **25.6%** | **0.002** | 92.6, 95% SI: (90.5, 94.4) | 72.1, 95% SI: (69, 75.7) | 2.1, 95% SI: (-15.8, 19.5) | 5.9, 95% SI: (5.2, 6.5) |
|  |  | **0.006** | 92.7, 95% SI: (90.7, 94.5) | 72.5, 95% SI: (69.3, 75.2) | 4, 95% SI: (-14.5, 21.2) | 5.8, 95% SI: (5.2, 6.4) |
|  |  | **0.009** | 92.8, 95% SI: (90.8, 94.6) | 72.9, 95% SI: (69.9, 76.3) | 4.3, 95% SI: (-14.6, 19.5) | 5.8, 95% SI: (5.2, 6.5) |
|  | **94.9%** | **0.002** | 97.2, 95% SI: (96, 98.3) | 80.1, 95% SI: (77.2, 82.8) | 8.3, 95% SI: (-7.4, 23.3) | 5.6, 95% SI: (5.1, 6.2) |
|  |  | **0.006** | 97.5, 95% SI: (96.3, 98.6) | 80.6, 95% SI: (77.8, 83.2) | 9.4, 95% SI: (-8.1, 24.5) | 5.6, 95% SI: (5.1, 6.1) |
|  |  | **0.009** | 97.8, 95% SI: (96.6, 98.8) | 81.1, 95% SI: (78.3, 84.1) | 9.8, 95% SI: (-6.8, 24.9) | 5.6, 95% SI: (5, 6.1) |

^a^ The TMP intervention consisted of HIV-1 RNA testing followed by standard rapid HIV tests to distinguish acute from prevalent infection. Newly diagnosed participants were immediately linked to care and offered PNS by a dedicated intervention team.

**Table S2. Sensitivity of model predictions to assumptions about the weekly probability of experiencing acute HIV symptoms and seeking treatment**

| Intervention | % of eligible patients being tested | Fraction of acute HIV infection seeking treatment | Percent diagnosed | Percent on Treatment | Percent of infection averted | HIV prevalence |
| --- | --- | --- | --- | --- | --- | --- |
| PITC at current rates | **28** | 69% | 90.7, 95% SI: (88.3, 92.8) | 67.5, 95% SI: (64.1, 70.6) | #N/A | 6.1, 95% SI: (5.4, 6.8) |
|  |  | 57.50% | 90.4, 95% SI: (88.3, 92.5) | 67.2, 95% SI: (63.6, 70.7) | #N/A | 6, 95% SI: (5.3, 6.6) |
| TMP^a^ | **28** | 69% | 92.7, 95% SI: (90.7, 94.5) | 72.5, 95% SI: (69.3, 75.2) | 4, 95% SI: (-14.5, 21.2) | 5.8, 95% SI: (5.2, 6.4) |
|  |  | 57.50% | 92.1, 95% SI: (90.1, 94.1) | 71.7, 95% SI: (68.5, 75.1) | 1.6, 95% SI: (-20.4, 19) | 5.9, 95% SI: (5.3, 6.5) |
|  | **90** | 69% | 97.5, 95% SI: (96.3, 98.6) | 80.6, 95% SI: (77.8, 83.2) | 9.4, 95% SI: (-8.1, 24.5) | 5.6, 95% SI: (5.1, 6.1) |
|  |  | 57.50% | 96.7, 95% SI: (95.4, 97.9) | 79.1, 95% SI: (76.1, 81.7) | 8.1, 95% SI: (-8.9, 23.2) | 5.6, 95% SI: (5.1, 6.2) |
